# Conformal Microfluidic‐Blow‐Spun 3D Photothermal Catalytic Spherical Evaporator for Omnidirectional Enhanced Solar Steam Generation and CO_2_ Reduction

**DOI:** 10.1002/advs.202101232

**Published:** 2021-08-07

**Authors:** Hao Liu, Hong‐Gang Ye, Minmin Gao, Qing Li, Zhiwu Liu, An‐Quan Xie, Liangliang Zhu, Ghim Wei Ho, Su Chen

**Affiliations:** ^1^ State Key Laboratory of Materials‐Oriented Chemical Engineering College of Chemical Engineering Jiangsu Key Laboratory of Fine Chemicals and Functional Polymer Materials Nanjing Tech University 5 Xin Mofan Road Nanjing 210009 P. R. China; ^2^ Department of Electrical and Computer Engineering National University of Singapore 4 Engineering Drive 3 Singapore 117583 Singapore; ^3^ CAS Key Laboratory of Carbon Materials Institute of Coal Chemistry Chinese Academy of Sciences Taiyuan 030001 China

**Keywords:** CO_2_ reduction, desalination, interfacial solar steam generation, microfluidic blow spinning, omnidirectional absorbance, photocatalysis

## Abstract

Solar‐driven water evaporation and valuable fuel generation is an environmentally friendly and sustainable way for clean water and energy production. However, a few bottlenecks for practical applications are high‐cost, low productivity, and severe sunlight angle dependence. Herein, solar evaporation with enhanced photocatalytic capacity that is light direction insensitive and of efficiency breakthrough by virtue of a three‐dimensional (3D) photothermal catalytic spherical isotopic evaporator is demonstrated. A homogeneous layer of microfluidic blow spun polyamide nanofibers loaded with efficient light absorber of polypyrrole nanoparticles conformally wraps onto a lightweight, thermal insulating plastic sphere, featuring favorable interfacial solar heating and efficient water transportation. The 3D spherical geometry not only guarantees the omnidirectional solar absorbance by the light‐facing hemisphere, but also keeps the other hemisphere under shadow to harvest energy from the warmer environment. As a result, the light‐to‐vapor efficiency exceeds the theoretical limit, reaching 217% and 156% under 1 and 2 sun, respectively. Simultaneously, CO_2_ photoreduction with generated steam reveals a favorable clean fuels production rate using photocatalytic spherical evaporator by secondary growth of Cu_2_O nanoparticles. Finally, an outdoor demonstration manifests a high evaporation rate and easy‐to‐perform construction on‐site, providing a promising opportunity for efficient and decentralized water and clean fuel production.

## Graphical abstract

A three‐dimensional (3D) photothermal catalytic spherical evaporator with double‐layer structure that enables the theoretical light‐to‐vapor efficiency limits breakthrough and efficient clean fuels production. The isotropy of spherical structure exhibits the omnidirectional light absorption, reducing the influence of natural light incident angle on solar evaporation rate and circumventing the trade‐off between water condensation and photocatalytic reaction, which is significant for practical applications.

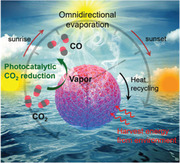

## 1 Introduction

Global demand of freshwater and clean energy is having significantly increased in the future for the rising population and social developments. As an inexhaustible energy source, solar energy can be harnessed and converted into various kinds of clean energy forms including electricity, chemical (fuel), and thermal energy, which is a promising way to provide solutions for clean water and energy scarcity with minimum environmental impact.^[^ ^1^, ^2^ ^]^ Recently, solar steam generation for sustainable freshwater production based on photothermic conversion technology has aroused tremendous research attention due to high photothermal conversion efficiency.^[^ ^3^, ^4^, ^5^ ^]^ The traditional solar evaporation methods based on bulk heating and expensive optical concentrator generally deliver a low light‐to‐heat conversion efficiency (30–45%) because of the poor sunlight absorption and large heat losses.^[^ ^6^, ^7^ ^]^ Most recently, solar‐driven interfacial evaporation by localization of solar heat to the air/liquid interface has been proposed to reduce thermal losses, and achieved evaporation efficiency of more than 90% under practical 1 sun.^[^ ^8^, ^9^ ^]^ However, compared with other conventional freshwater production technologies, like reverse osmosis and multi‐stage flash, the water evaporation flux through solar steam generation under natural environment is lower due to the limitation of natural solar irradiation energy intensity (1 kW m^−2^).^[^ ^10^, ^11^ ^]^ Assuming 100% solar‐to‐vapor energy conversion efficiency, the upper limit of evaporation rate is only 1.68 kg m^−2^ h^−1^ without thermal recycling.^[^ ^12^ ^]^ Therefore, it would be intriguing and significant for practical utilization to break through this evaporation rate/efficiency limit. To date, two pathways/mechanisms have been presented to realize this purpose. One is reducing water vaporization enthalpy by the intrinsic molecular meshes of a hydrogel.^[^ ^13^, ^14^, ^15^ ^]^ The other method is harvesting extra energy from the environment through evaporator structural designs, including cylindrical,^[^ ^16^ ^]^ cup‐shaped^[^ ^17^ ^]^ and triangle structures.^[^ ^12^ ^]^ Due to the evaporative cooling and less solar energy absorption, the temperature of the side/partial surfaces of these evaporators decrease below the environment temperature, thus allow them to harvest energy from the warmer environment, resulting in a limit‐breaking vaporization efficiency.^[^ ^16^ ^]^ However, this breakthrough technique is generally realized under low‐intensity solar illumination (≤1 sun). Considering the high‐intensity solar illumination would elevate the temperature of the entire evaporator, hence reduce the temperature difference and even reverse the heat transfer direction. This directly decreases/eliminates the environmental enhancement effect, hindering massive freshwater production by solar evaporation.^[^ ^16^, ^18^ ^]^ Similarly, the reduction of CO_2_ with solar energy and water, has been considered to be the most attractive solution to convert greenhouse gas into valuable chemicals or fuels. Importantly, the photothermal effect serves as an enhancement pathway for photocatalysis through extending the light absorption range of catalysts beyond their bandgap to promote solar‐to‐chemical energy conversion. Nevertheless, it is still rare for synergistic freshwater production or clean fuel generation by solar photothermal phenomenon, so as to effectively harvest and utilize whole solar spectrum.^[^ ^4^ ^]^

On the other hand, solar azimuth and elevation angle are ever varying. Maximum light absorbance and conversion of solar absorber materials are generally achieved when they are tilted to face the sun perpendicularly.^[^ ^19^ ^]^ Nevertheless, expensive and complicated mechanical tracking infrastructures that require high capital investment and energy consumption are normally employed to ensure accurate and continuous angle manipulation. This seriously impedes the development and actual disposition at off‐grid and remote regions. Therefore, solar converting materials with fixed, uncomplicated structural design, devoid of ancillary moveable parts which has efficient sunlight absorption for most part of the day are essential.^[^ ^20^ ^]^

Herein, we report a high‐performance three‐dimensional (3D) photothermal catalytic spherical evaporator via microfluidic blow spinning (MBS) technology to realize a new concept of synergetic system for photothermic‐enhanced freshwater production and valuable chemicals generation. The emerging MBS technique is safe, productive, and easy‐to‐manipulate the microstructure of nanofibers (NFs), having been regarded as a most promising NF fabrication technology for industrialization. The 3D spherical evaporator is fabricated by conformally spinning polyamide 6,6 (PA66) NFs onto a lightweight plastic sphere, followed by an in situ growth of polypyrrole (PPy) solar absorber, endowing the evaporator with a large specific surface area and interconnected fibrous network structure so as to enhance light absorption and water wettability. As a result, a theoretical limit breakthrough in the light‐to‐vapor efficiency is achieved, which reaches up to ≈260% and 180% under respective light intensity of 1 and 2 kW m^−2^, leveraging on the environmental energy harnessing from the non‐illuminated region. The photoreduction of CO_2_ with generated vapor achieves the favorable chemical fuels production using photocatalytic spherical evaporator by secondary growth of Cu_2_O photocatalyst. Importantly, the isotropy of spherical structure exhibits an omnidirectional light absorption behavior, in contrast to planar evaporators that require constant adjustment to receiving incident sun ray perpendicularly. Moreover, the characteristic of spherical structure maintains the lower part of the sphere under shadow of lower surface temperature to favorably gain energy through latent heat recycling and ambient energy harvesting, achieving high evaporation, invariant of light irradiation directions. In this way, it significantly reduces the influence of natural sunlight incident angle on evaporation rate, and further extends the effective evaporation period during the daytime. Meanwhile, oblique incident light circumvents the upper condensate droplets that severely block the light absorbance by reflection and scattering, which dramatically enhances the photocatalytic CO_2_ reduction performance, simultaneously promoting the solar comprehensive utilization and steam condensation. Finally, a solar still assembled with modular based spherical evaporator units is demonstrated, resulting in a high solar‐to‐vapor efficiency of 195% at outdoor conditions. Therefore, the low‐cost, energy‐effective, scalable, and portable photothermal catalytic spherical evaporator may offer a good alternative construct for freshwater and clean energy supply in off‐grid/remote areas.

## 2 Results and Discussion

**Figure 1:**
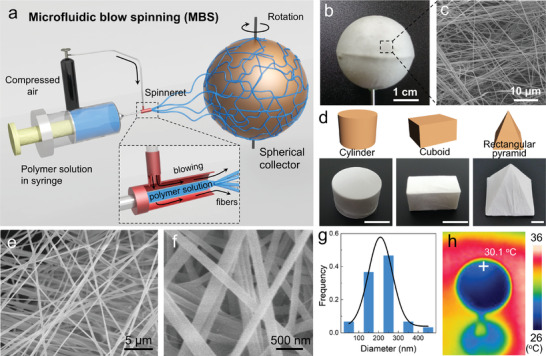
a) Schematic illustration of preparation of PA66 sphere via MBS. b) Photograph of PA66 sphere. c) SEM image of the PA66 NFs sphere. d) Schematic of 3D collectors with different geometric features and corresponding photographs after conformal deposition of PA66 NFs, scale bar: 1 cm. e,f) SEM images of blow spun PA66 NFs under the flow rate of 5 mL h^−1^, the air pressure of 0.1 MPa, the concentration of spinning solution of 13%, and the collection distance of 35 cm. g) Fiber diameter distribution of PA66 NF film. h) IR image of PA66 sphere after 30 min light irradiation.

The PA66 sphere is prepared by a MBS methodology. The blow spinning method has been used to fabricate the polymeric ultrafine NFs, which is not only free from the high voltages and metallic collectors but also safe, productive, and easy‐to‐operate.^[^ ^21^, ^22^, ^23^ ^]^ Moreover, microfluidic technique is emerging as a powerful tool to process and manipulate the micro‐droplet/fluid within the microtubes or microfluidic chips, producing various microstructural nanomaterials, for example, microbeads, microcapsules, Janus particles, and fibers.^[^ ^24^, ^25^, ^26^, ^27^, ^28^ ^]^ Introducing microfluidic technology into the blow spinning process could realize a precise control of the spinning solution.^[^ ^29^, ^30^, ^31^ ^]^ Typically, steady airflow behind the tip of spinneret where PA66 solution is regulated in a microtube drags the PA66 solution into fine NFs, and then deposits them on a revolving target collector, which in this case is a 3D plastic sphere (**Figure** **1a**). As a result, blow spun PA66 NFs are uniformly deposited on the sphere collector to form a conformal spherical white membrane of continuous fibrous networks (Figure 1b,c). Notably, deposition of PA66 NFs based on MBS technique is also attempted on other 3D collectors with different geometric features, for example, cylinder, cuboid, and rectangular pyramid, resulting in a highly compliant and homogenous coatings (Figure 1d). As shown in Figure 1e–g, the smooth PA66 NFs with an average diameter of ≈200 nm can be facilely produced. The influences of the experimental parameters including the compressed air pressure, flow rate, the concentrations of the spinning solution on fiber morphology, and diameter were studied in detail (Figures S1–S3, Supporting Information). In general, higher air pressure and lower flow rate lead to smaller fiber diameter.

**Figure 2:**
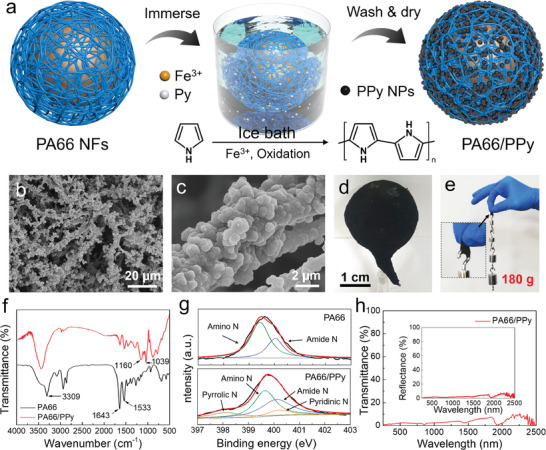
a) Schematic of the in situ synthesis of PPy on PA66 NFs in an ice bath. b,c) SEM images of PA66/PPy NFs. d) Photograph of PA66/PPy sphere. e) Photographs of weights hooked by a piece of PA66/PPy NF film (film size: 2 cm × 1 cm). f) FTIR and g) XPS spectra of PA66 and PA66/PPy NFs. h) Transmittance and reflectance spectra of PA66/PPy NF film.

However, due to the low light absorption, the pure PA66 fibrous sphere cannot meet the requirements of highly efficient solar evaporator (Figure 1h; Figure S4, Supporting Information). PPy is one of the most effective solar absorbers, which could be coated on the fibrous membrane in situ to achieve a high light‐to‐thermal conversion efficiency.^[^ ^13^ ^]^ Meanwhile, PA66 sphere has an interconnected fibrous network architecture, which provides an ideal scaffold for the immobilization of photothermal materials and water transport pathways. By immersing into pyrrole (Py) monomer solution in an ice bath, PPy nanoparticles (NPs) were polymerized and coated on the surface of NFs (**Figure** **2a**; Figure S5, Supporting Information). The SEM images in Figure 2b,c show that the PPy NPs are distributed along the blow spun PA66 NFs, improving the roughness of the spun film. The corresponding digital photograph of PA66/PPy sphere is shown in Figure 2d, displaying a color change from white to black. A thin strip of PA66/PPy NF film (2 cm × 1 cm) can easily hold up weights above 180 g without giving way (Figure 2e), indicating its excellent tensile strength. Furthermore, the PA66 sphere loaded with different amount PPy (based on the concentration of Py monomer in solution: 0.001, 0.01, 0.05, 0.1, and 0.2 mol L^−1^) are referred as PA66/PPy_0.001_, PA66/PPy_0.01_, PA66/PPy_0.05_, PA66/PPy_0.1_, and PA66/PPy_0.2_, respectively. SEM images show increase PPy NPs loading with higher concentration of Py (Figure S6, Supporting Information). At low concentration of 0.001 mol L^−1^, sparsely distributed PPy NPs are observed on the surface of the PA66 NFs (Figure S7a, Supporting Information). When the Py concentration is increased to 0.2 mol L^−1^, PPy NPs are shown to cover the entire PA66 film (Figure S7b, Supporting Information).

The chemical compositions of PA66 NFs and PA66/PPy composites were confirmed by FTIR. As presented in Figure 2f, the peak at 3309 cm^−1^ is ascribed to vibrations of N—H stretching. The peaks of 1533 and 1643 cm^−1^ are assigned to the vibration of amide I and amide II bands in PA66 NFs.^[^ ^32^ ^]^ Compared with PA66 NFs, the new peaks at 1160 and 1039 cm^−1^ in PA66/PPy composite correspond to stretching vibrations of C—O—C in the PPy NPs, indicating PPy NPs have been assembled onto PA66 NFs.^[^ ^33^ ^]^ XPS spectra in Figure 2g and Figure S8, Supporting Information show the peaks at 284.5, 400, and 528 eV attribute to C1s, N1s, and O1s of PA66 and PA66/PPy. The C1s spectrum of PA66 can be deconvoluted into three peaks at 284.3 (C═C), 285 (C—C), and 287.7 eV (C═O). After PPy growth, the peaks of C1s shift to 284.6, 286.4, and 287.6 eV.^[^ ^34^ ^]^ In the N1s spectrum of PA66, it can be fitted into two peaks 399.4 (amino nitrogen) and 400 eV (amide nitrogen). The N1s spectrum of PA66/PPy can be fitted into four peaks at 398.3 (pyrrolic nitrogen), 399.6 (amino nitrogen), 400.1 (amide nitrogen), and 400.2 eV (pyridinic nitrogen).^[^ ^35^, ^36^ ^]^ XRD analysis shows that the characteristic peaks of PA66 NFs appear at 20° and 24°, indicating the *α* phase of PA66 (Figure S9, Supporting Information).^[^ ^37^ ^]^ An obvious increment at 2*θ* = 23° in PA66/PPy suggests the introduction of PPy.^[^ ^35^ ^]^ All these characterizations revealed that PPy NPs have been successfully loaded on PA66 NF film, resulting in carbon and nitrogen‐rich surface which could enhance the light absorption and water wettability.^[^ ^36^ ^]^

**Figure 3:**
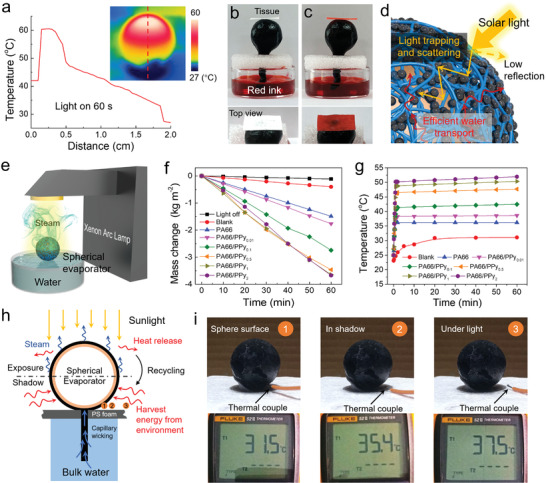
a) IR image of PA66/PPy sphere after 1 min light irradiation and the temperature profile of the marked dash line in the IR image. b,c) Photographs of tissue paper placed on the top of PA66/PPy sphere before and after wicking red ink water. d) Schematic illustration of photothermal conversion on evaporator surface. e) Schematic diagram of the solar steam generation. f) Evaporation mass changes and g) surface temperature changes of spherical evaporators with different PPy loading under an optical density of 1 kW m^−2^. h) Schematic mechanism of heat flows in photothermal evaporation process based on spherical evaporator. i) Photographs of evaporator and corresponding temperatures on the surface of lower hemisphere, in the shadow and ambient environment.

The PA66/PPy NF film with a thickness of 0.5 mm displays low optical transmittance (≈0.5–7%) and reflectance (≈0.5–5%) across the full solar spectrum range (250–2500 nm) (Figure 2h). This signifies superior solar absorption pertaining to the optical absorption of PPy NPs and the light scattering in the interconnected fibrous network which increases the optical transfer path length. Moreover, the unique double‐layer configuration consisting of the conformal spherical PA66/PPy film serving as a confined 2D water path that is wrapped on a hollow plastic sphere to mitigate heat losses.^[^ ^38^ ^]^ The high optical absorbance capability and low heat loss of PA66/PPy sphere make it a potential evaporator for solar steam generation with high photothermal efficiency. As shown in **Figure** **3a**, the temperature of the upper hemisphere surface of the spherical evaporator can reach more than 60 °C within 1 min under 1 kW m^−2^, while the lower hemisphere surface maintains a lower temperature. The temperature profile exhibits a localized sharp peak, implying trapping of photothermal heat. In addition, interconnected fibrous network with high wettability provides the pathways for both water wicking to the surface and subsequent steam escape. Evidently, the water droplet readily absorbed in 0.2 s, which is attributed to high wettability of PPy (Figure S10, Supporting Information). In contrast, the contact angle of pure PA66 film is measured to be 108° (Figure S11, Supporting Information). A capillary wicking of red‐ink water was also carried out to show that the white tissue placing on the top of PA66/PPy sphere is stained red, suggesting an effective water transportation (Figure 3b,c). Collectively, rough surface of blow spun PA66/PPy sphere not only plays a great role for efficient water transportation, but also enhances the solar absorptance by trapping/scattering of incident light and reducing direct single reflections (Figure 3d).

Due to its excellent light absorption and water transport properties, PA66/PPy sphere was employed as the spherical evaporator to enable interfacial water evaporation under solar irradiation (Figure 3e). A spherical evaporator on a PS foam with a tail structure contacting with bulk water for continuous water wicking was prepared (Figure S12, Supporting Information). Rapid light absorption and vapor generation occur at the spherical surface. Spherical evaporators of 2.5 cm diameter with different PPy NP loading were tested for solar steam generation. The evaporation rates of the PA66/PPy spherical evaporators were quantified by recording the weight loss of water, which are 1.48, 1.76, 2.74, 3.46, 3.64, and 3.67 kg m^−2^ h^−1^ for PA66, PA66/PPy_0.001_, PA66/PPy_0.01_, PA66/PPy_0.05_ PA66/PPy_0.1_, and PA66/PPy_0.2_ (Figure 3f). The corresponding light‐to‐vapor conversion efficiencies (*η*) are calculated to be 75.4%, 90.2%, 140%, 177%, 186%, and 187% (Equations S1–S3, Supporting Information), which surpass the theoretical efficiency limit.^[^ ^16^ ^]^ Among the different PPy NPs loadings, the PA66/PPy_0.1_ evaporator shows a similar evaporation performance as the PA66/PPy_0.2_ evaporator, suggesting the optimized loading amount is achieved in 0.1 mol L^−1^ Py monomer solution. Moreover, the temperature at the top of the PA66/PPy_0.1_ spherical evaporator is 52 °C, which is 20 °C higher than that of blank water (Figure 3g). Notably, a stable evaporator surface temperature could be reached within 1 min (Figure S13, Supporting Information), revealing that confined 2D water path could localize the solar heat at the surface of the evaporator while the inner hollow plastic sphere functions as a good thermal insulator, so as to minimize heat loss and augment photothermal conversion. Solar irradiation from the top, middle, and bottom of the spherical evaporator could realize higher than 100% light‐to‐vapor efficiency under 1 sun (Figure S14, Supporting Information)^.[^ ^39^ ^]^ Furthermore, the spherical evaporator is mechanically stable and robust for reusability and recycling without obvious deterioration of water evaporation ability (Figure S15, Supporting Information). It is apparent that a prudently designed 3D PA66/PPy spherical evaporator dramatically enhances the solar evaporation rate and light‐to‐steam efficiency to more than 100% theoretical limit, which is also higher than most of other 3D structural designs^.[^ ^16^, ^17^ ^]^

To illustrate how this can be achieved, Figure 3h,i depicts the mechanism of high light‐to‐vapor efficiency. It can be seen that the upper hemispherical surface of the evaporator absorbs most of incident solar energy to trigger water evaporation, which leads to high surface temperature of 52 °C. In a closed system, the latent heat in vapor is subsequently released to the environment during the evaporation. Together with the heating action of light that does not shine on the 3D PA66/PPy spherical evaporator, the internal temperature of the system is improved. On the other hand, due to the shadowing of the upper hemisphere, the lower hemispherical surface of the evaporator cannot absorb the solar energy directly. In the meantime, natural evaporative cooling occurs, reducing the surface temperature of the selective shadowed evaporator (Figure 3h). It is experimentally verified that the temperature of the lower hemisphere, shadow, and the immediate surrounding ambient are 31.5, 35.4, and 37.5 °C, respectively (Figure 3i), revealing that the surface temperature of the non‐illuminated hemisphere is much lower than that of the surrounding environment (37.5 °C). Therefore, the lower hemisphere can harvest energy from the environment by convective and radiative effects. The evaporation rate beyond the theoretical limit can be realized by synchronous minimization of the energy loss from the top surface and maximization of the energy gain from the lower surface through latent heat recycling and environmental energy harvesting. Compared with other 3D evaporators, the light‐to‐vapor conversion efficiency is higher due to the presence of shadow in the spherical evaporator design.^[^ ^16^, ^17^ ^]^

**Figure 4:**
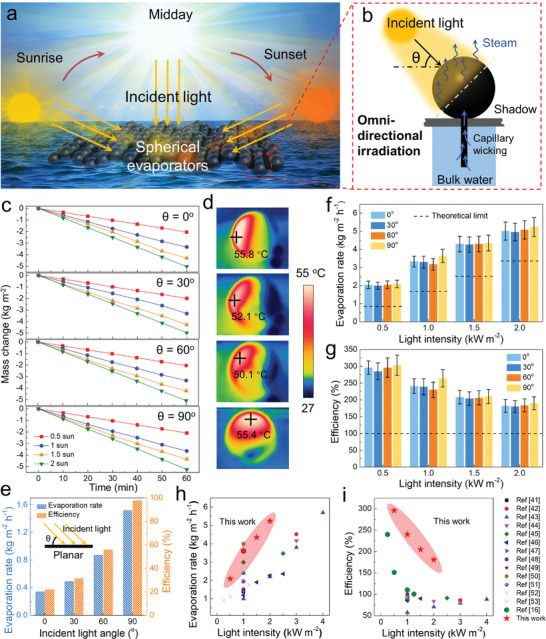
a) Schematic illustration of the spherical evaporator under sunrise and sunset. b) Schematic illustration of the spherical evaporator under different incident angles of light. c) Evaporation mass loss over time under different solar intensities (0.5, 1, 1.5, and 2 kW m^−2^) at different light angles of 0°, 30°, 60°, and 90°. d) Infrared images of the spherical evaporator at different illumination angles under light intensity of 1 kW m^−2^. e) Evaporation rate and light‐to‐vapor efficiency of the planar evaporator at different illumination angles under light intensity of 1 kW m^−2^. f) Evaporation rate and g) light‐to‐vapor efficiency of spherical evaporator under different light angles and light intensities. Comparison of solar h) evaporation rate and i) light‐to‐vapor efficiency obtained from highly related publications and spherical evaporator.

**Figure 5:**
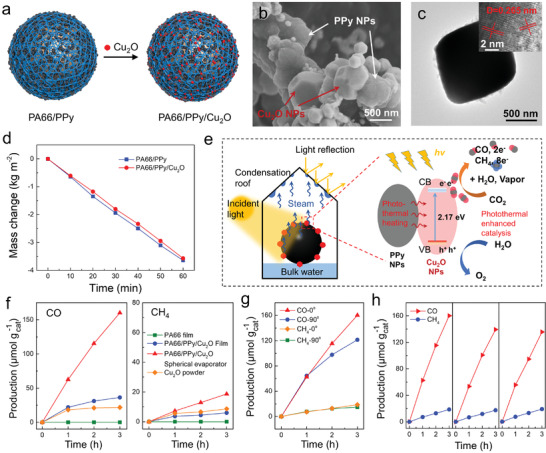
a) Schematic of synthesis of PA66/PPy/Cu_2_O photocatalytic evaporator. b) SEM image of PA66/PPy/Cu_2_O NFs. c) TEM image of Cu_2_O NPs. Inset: HRTEM image of Cu_2_O. d) Evaporation mass changes of PA66/PPy and PA66/PPy/Cu_2_O spherical evoprators under solar intensity of 1 kW m^−2^. e) Schematic diagram of the photothermal enhanced catalytic CO_2_ reduction by photocatalytic PA66/PPy/Cu_2_O evaporator. f) CO and CH_4_ evolution by CO_2_ photoreduction as a function of light irradiation time using different photocatalysts. g) CO and CH_4_ evolution by the spherical PA66/PPy/Cu_2_O evaporator under different incident angles of light (vertical and horizontal). h) Multiple cycles of CO and CH_4_ production by photocatalytic PA66/PPy/Cu_2_O evaporator.

The solar azimuth and elevation angle vary throughout the day. The conventional 2D steam generators cannot absorb all the rays so long as it is at non‐perpendicular incidence, reducing the acquired photon density from the sun. Whereas, the isotropic spherical evaporator developed in this work could always keep a hemispherical surface facing the sunlight despite the light incident angle, assuring the omnidirectional solar absorbance for solar evaporation all day long (**Figure** **4a**).^[^ ^40^ ^]^ The solar simulator from perpendicular to the horizontal directions were modulated to change the angle of light (*θ* = 90°, 60°, 30°, and 0°, Figure 4b; Figure S16, Supporting Information). The results show that the average water evaporation rates are 3.17, 3.09, and 3.33 kg m^−2^ h^−1^ under solar angles of 60°, 30°, and 0° at 1 sun illumination, which are similar to that under 90° (3.6 kg m^−2^ h^−1^) (Figure 4c). The corresponding surface temperatures are higher than 50 °C (Figure 4d). In contrast, planar film evaporator using the same material displayed a dramatic reduction in evaporation rate with the decreasing incident angles, from 1.52 to 0.34 kg m^−2^ h^−1^ (Figure 4e). The corresponding light‐to‐vapor efficiencies drastically decrease from 78% to 17%. It is noted that the high evaporation rate is also achieved under different light intensities. As shown in **Figure** **5c**,f,g and Tables S2,S3, Supporting Information, at lower light intensities, the efficiencies reach up to ≈250%. Even when the light intensity is improved to 2 kW m^−2^, it still guarantees an efficiency limit breakthrough, that is, ≈150%, due to the 3D spherical structure and the perpetual shadowing (Figure 4f). To evaluate the results of this work, recent related publications are summarized and presented in Figure 4h,i, which clearly show that both the evaporation rate and efficiency manifested by as‐prepared isotropic spherical evaporator are higher than most of the reported works.^[^ ^16^, ^41^, ^42^, ^43^, ^44^, ^45^, ^46^, ^47^, ^48^, ^49^, ^50^, ^51^, ^52^, ^53^ ^]^ In a nutshell, by the rational 3D structural design of a spherical evaporator could greatly enhance the solar evaporation rate and efficiently utilize the solar light. It retains a high light‐to‐vapor efficiency under elevated light intensity, which is superior to column‐shape evaporators that are only effective for ambient energy harvesting under low solar power density. More importantly, as the majority of the solar energy is in the direct rather than diffuse beam, maximizing light irradiance through the omni‐directional light absorbance spherical evaporator circumvents the influence of natural light incident angle, favoring practical applications.

Additionally, solar photothermal effect serves as an enhancement strategy for photocatalysis could significantly promote solar‐to‐chemical energy conversion.^[^ ^54^, ^55^, ^56^ ^]^ By in situ secondary growth of semiconducting photocatalyst Cu_2_O NPs, the spherical evaporator is endowed with supplementary photocatalysis ability, capable of photoreducting CO_2_ into valuable chemicals of CO and CH_4_ (Figure 5a). The SEM image of PA66/PPy/Cu_2_O reveals the cross‐distribution of PPy and Cu_2_O NPs along the PA66 NF (Figure 5b). The TEM image shows an individual quasi‐cubic Cu_2_O NPs with the lattice spacing is 0.278 nm which corresponds to the (111) plane of Cu_2_O (Figure 5c).^[^ ^57^, ^58^ ^]^ The FT‐IR spectra show a sharp absorption peak at 635 cm^−1^ (Cu—O) and XRD spectra with new diffraction peaks at 36.1°, 42.5°, 62.3°, and 74.3° are attributed to the Cu_2_O NPs (Figures S17,S18, Supporting Information), manifest the successful growth of P‐type Cu_2_O. Compared with PA66/PPy evaporator, only a slight degradation on solar evaporation rate occurs after Cu_2_O loading, suggesting a negligible influence of photocatalyst on purified water yield (Figure 5d). Typically, semiconductor Cu_2_O can be excited by incident light with the electrons transferred from valence band (VB) to conduction band, leaving behind holes at the VB. The generated electrons serve as the active sites for CO_2_ reduction (Figure 5e). Significantly, the PPy NPs can effectively broaden the light absorption range of PA66/PPy/Cu_2_O NFs due to their full‐spectrum light absorption properties to produce a local heat source. Instanteneously, the photoheat is delivered to the photocatalytic active sites on adjacent Cu_2_O, enhancing the activity of electron–hole pairs. Together with the pyrrolic nitrogen atoms in PPy NPs, they efficiently adsorb and concentrate CO_2_ molecules, boosting photocatalytic reactions.^[^ ^9^, ^59^ ^]^ As a result, the evolution rates of CO and CH_4_ from CO_2_ photoreduction with generated vapor are 62.55 and 7.21  µmol g_cat_^−1^ h^−1^ by the PA66/PPy/Cu_2_O photocatalytic spherical evaporator, respectively, which are highly improved compared with those based on catalysts of pure Cu_2_O powder (18.19 and 5.51 µmol g_cat_^−1^ h^−1^ for CO and CH_4_ production, respectively) and PA66/PPy/Cu_2_O planar evaporator (21.55 and 3.66 µmol g_cat_^−1^ h^−1^ for CO and CH_4_ generation, respectively) (Figure 5f). It is experimentally verified that the Cu_2_O/PA66 sphere without PPy loading exhibits the production rates of CO and CH_4_ are 20.43 and 5.42 µmol $g_{\mathrm{cat}}^{-1}$ h^−1^, which are much lower compared with PA66/PPy/Cu_2_O sphere, revealing the photocatalytic enhancement by photothermal effect (Figure S19, Supporting Information). It should also be noted that a more stable CO and CH_4_ production rates are achieved under horizontal light irradiation, in contrast to an obvious decrease after 1 h when the light is illuminated from the top (Figure 5g), which is attributed to the vapor condensate droplets on the roof/top of the quartz reactor that severely block the light due to reflection and scattering. The oblique light evades the upward pathway of vapor, dramatically increasing the incidence of the light and enhancing photocatalytic CO_2_ reduction performance. The reusability of PA66/PPy/Cu_2_O photocatalytic spherical evaporator for CO and CH_4_ evolution is also demonstrated in Figure 5h. In a nutshell, the rational design of the 3D photothermal catalytic spherical evaporator could circumvent the trade‐off between water condensation and photocatalytic performance, synergistically realizing the efficient freshwater and clean fuel production by effectively harvesting and utilizing the whole solar spectrum.

**Figure 6:**
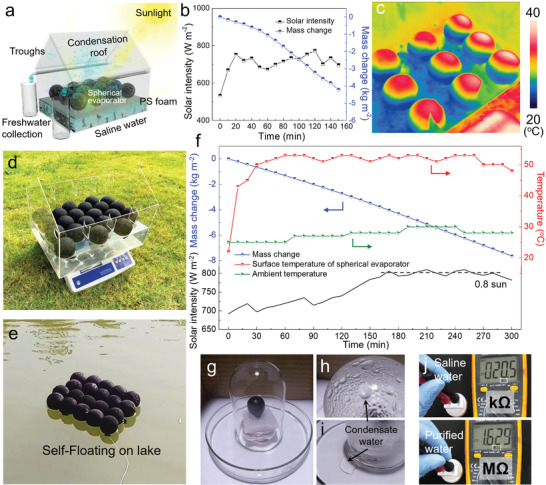
a) Schematic diagram of a model house solar evaporator equipping with condensation roof, water troughs, and collection bottles. b) Solar intensity and evaporation mass loss of solar evaporator assembled in quadrate configuration outdoors from 10:30 am to 1 pm on June 26, 2020 at Nanjing Tech University, Jiangpu campus. c) IR image of the solar still under natural irradiation. Photographs of assembled spherical evaporators self‐floating on d) the model house prototype and e) the lake. f) Solar intensity spectrum, evaporation mass loss, and synchronous ambient and surface temperature changes outdoors from 10 am to 4 pm on September 11, 2019 at the same venue. g–i) Photographs of vapor condensation and water collection after 1 h light irradiation. j) Evaluation of water purity by a multimeter with a constant distance between electrodes.

Finally, to explore the feasible application of spherical solar evaporator, a simple prototype of model solar still with size of 40 cm × 30 cm × 35 cm was fabricated to perform a solar desalination using the photothermal spheres under natural light conditions.^[^ ^36^, ^60^ ^]^ A larger PA66/PPy spheres with diameter of 7 cm were prepared and orderly arranged in different configurations on the water surface in the solar still (quadrate and hexagonal arrangement configurations, Figure S20, Supporting Information). Based on this prototype, further vapor condensation and freshwater collection can be achieved by equipping with condensation roof, water troughs, and collection bottles, which are designed and illustrated in **Figure** **6a**. In the quadrate arrangement configuration, PA66/PPy spheres are placed on a PS foam board with tails structure contacting with bulk water (Figures S20a,S21, Supporting Information). Figure 6b shows the natural solar intensity and water mass loss of PA66/PPy spheres array in outdoor conditions (from 10:30 am to 1 pm on June 26, 2020). Accordingly, the evaporation rate and light‐to‐vapor efficiency are calculated to be about 2.21 kg m^−2^ h^−1^ and 160% under an average solar intensity of 0.71 sun, which are lower than that conducted in the laboratory, but still exceed 100%. The corresponding IR image in Figure 6c shows a higher temperature of the upper hemisphere, indicating good heat localization. In addition, owing to the lightweight attribute, the spherical evaporator unit can be facilely assembled in the hexagonal arrangement configuration on a large scale and float on the lake surface directly, without necessitating extra floating base and insulating materials, facilitating the solar still construction (Figure 6d,e; Figures S20b,S22, Supporting Information). Figure 6f shows the evaporation performance of PA66/PPy spheres in the hexagonal arrangement configuration on a sunny day (from 10 am to 4 pm on September 11, 2019). The natural solar intensity increased gradually in the morning, maintained at 0.8 sun in the middle day and decreased in the afternoon (Figure 6f, black). The ambient and evaporator surface temperatures are consistent with the trend of solar intensity (Figure 6f, red and olive). The surface temperature of spherical solar evaporator quickly rose to ≈54 °C due to the rapid interfacial heating characteristics of the spherical evaporator, which is more than 24 °C higher than ambient temperature. The average water evaporation rate is 1.6 kg m^−2^ h^−1^ with an effective area of 0.077 m^2^ (Figure 6f, blue) under 0.8 kW m^−2^. The corresponding solar‐to‐vapor efficiency is ≈106.6%, which is lower than that of PA66/PPy spheres in the quadrate arrangement configuration. This decrease is due to the fact that the bottom of spherical evaporators is immersed in bulk water, thus reduces the environmental energy harvesting while the compact arrangement of spheres impedes light absorbance and vapor escape (Figure S23, Supporting Information). Notably, the water mass change shows continuous decrease regardless of the position of the sun and light incident angle. With a transparent glass cover, the vapor is successfully condensed into water droplets and collected in a beaker (Figure 6g–i). A layer of mist was formed on the glass cover in 5 min by spherical evaporator, in contrast to planar evaporator which required 10 min, indicating the rapid vapor generation in spherical evaporator system (Figure S24, Supporting Information). As a result, the average water collection rate is 2.21 kg m^−2^ h^−1^ (Table S4, Supporting Information). More importantly, the outdoor daily water collection rate is ≈18 kg m^−2^ under the average light intensity of 0.5 kW m^−2^ (Table S5, Supporting Information). The amount of collected water could meet the daily drink water demand of a family, which is competitive with other similar works.^[^ ^61^, ^62^ ^]^ The resistance values of 0.02 and 1.6 MΩ of saline water and purified water are obtained from a resistance measurement by a multimeter at constant distance between electrodes, suggesting effective desalination of seawater (Figure 6j).^[^ ^13^ ^]^ Considering the low‐cost and portability, the spherical solar evaporator shows practical feasibility of high‐performance vaporization under natural outdoor conditions, especially valuable for remote or emergency drinking water supply.

## 3 Conclusion

In summary, we have demonstrated a highly efficient photothermal catalytic spherical evaporator by conformally MBS of PA66 NFs onto a spherical collector with subsequent in situ growth of PPy NPs light absorber and Cu_2_O photocatalyst. The blow spun NFs impart a high specific surface area and interconnected fibrous network to enhance light absorption and water transportation. The elaborate 3D spherical geometry not only guarantees the omnidirectional solar absorbance by the light‐facing hemisphere, but also keeps the other hemisphere under shadow to harvest the energy from warmer environment, giving rise to the light‐to‐vapor efficiency of 217% and 156% under 1 and 2 sun, respectively. Moreover, the photoreduction of CO_2_ with solar steam achieves a favorable CO production rate of 62.55 µmol g_cat_^−1^ h^−1^ by photocatalytic spherical evaporator after secondary growth of Cu_2_O photocatalyst. The omnidirectional light absorption increases the tolerance toward ever changing sunlight incident angle for evaporation and photocatalysis, maximizing light harnessing for as long as possible, in contrast to the 2D photocatalytic planar evaporators. Finally, localized photothermal steam generation under unstructured outdoor condition with a high solar‐to‐vapor efficiency of 160% is demonstrated. This work shows that the cost‐and energy‐effective photothermal catalytic spherical evaporator may provide an alternative conception for emergent decentralized water and clean fuel supply for off‐grid/remote areas.

## Conflict of Interest

The authors declare no conflict of interest.

## Supporting information

Supporting Information

## Data Availability

The data that supports the findings of this study are available in the supplementary material of this article.
