# Supplementary material for: Conformal Microfluidic‐Blow‐Spun 3D Photothermal Catalytic Spherical Evaporator for Omnidirectional Enhanced Solar Steam Generation and CO2 Reduction
Source: Adv Sci (Weinh). 2021 Aug 7;8(19):2101232. doi: 10.1002/advs.202101232 (PMC8498876; doi:10.1002/advs.202101232)
Supplement: Supplementary file 1 — Supporting Information [file ADVS-8-2101232-s001.pdf]

WILEY-VCH

© 2021 Wiley-VCH GmbH

ADVANCED SCIENCE
Open Access

Supporting Information

for *Adv. Sci.*, DOI: 10.1002/advs.202101232

Conformal Microfluidic-Blow-Spun 3D Photothermal Catalytic Spherical Evaporator for Omnidirectional Enhanced Solar Steam Generation and CO_2 Reduction

Hao Liu, Hong-Gang Ye, Minmin Gao, Qing Li, Zhiwu Liu, An-Quan Xie, Liangliang Zhu, Ghim Wei Ho* and Su Chen**

Supporting Information

Conformal Microfluidic-Blow-Spun 3D Photothermal Catalytic Spherical Evaporator for Omnidirectional Enhanced Solar Steam Generation and CO_2 Reduction

Hao Liu, Hong-Gang Ye, Minmin Gao, Qing Li, Zhiwu Liu, An-Quan Xie, Liangliang Zhu, Ghim Wei Ho* and Su Chen**

1. Experimental Section

Materials

Polyamide 6,6 (PA66, M_w = 262.35 g mol^{-1}) was purchased from Aldrich. Ferric chloride ($FeCl_3$), pyrrole (Py) and sodium chloride (NaCl) were purchased from Aladdin. Formic acid (≥ 88%, analytical reagent) was purchased from Sinopharm Chemical Reagent Co., Ltd. All chemicals were used as received without any further purification. Deionized (DI) water with resistance greater than 18 MΩ cm^{-1} was used in all experiments.

Preparation of PA66 Sphere by Microfluidic Blow Spinning

13 g of PA66 powder was dissolved in 87 g of formic acid to obtain 13 wt% PA66 spinning solution. Loaded PA66 spinning solution in a 20 mL syringe which manipulated by a microfluidic pump. The hollow plastic sphere with diameter of 2.5 cm was used as a fiber collector. PA66 NFs was microfluidic blow spun to collect on the plastic sphere with rotation at a proper speed under the microfluidic flow rate of 5 mL h^{-1}, air pressure of 0.1 MPa and collection distance of 35 cm. The PA66 planar film was prepared using a flat nylon mesh as collector under the same spinning conditions.

In-situ Preparation of PA66/PPy Spheres

1.4 g of $FeCl_3$ was dissolved in 150 g of DI water. The as-spun PA66 sphere was immersed in $FeCl_3$ solution for 0.5 h, subsequently 0.01, 0.1, 0.5, 1 and 2 g of Py was added into the solution in ice bath and reacted for 6 h. The obtained spheres were recorded as PA66/$PPy_{0.001}$, PA66/$PPy_{0.01}$, PA66/$PPy_{0.05}$ PA66/$PPy_{0.1}$ and PA66/$PPy_{0.2}$. After reaction, the PA66/PPy spheres were washed by DI water for 3 times and dried at 50 ^{o}C. The PA66/PPy film was prepared under in monomer Py concentration of 0.1 mol L^{-1}.

In-situ Preparation of PA66/PPy/Cu2O Photocatalytic Spherical Evaporator

2 g of $CuSO_4$ and 1.2 g of NaOH were dissolved in 50 and 30g of DI water, respectively. The as-spuncwas immersed in $CuSO_4$ solution for 0.5 h, followed by adding NaOH solution dropwise into the $CuSO_4$ solution to form $Cu(OH)_2$ NPs on PA66/PPy sphere. 1.2 g of D-glucose was then added in the mixture under 70 ^{o}C for 2.5 h to reduce $Cu(OH)_2$ to Cu_2O. The pure Cu_2O NPs were synthesized under the same condition in absence of PA66/PPy sphere.

Characterization

The morphology of the as-spun NFs was observed by scanning electron microscope (SEM) with a QUANTA 200 (Philips-FEI, HOLLAND) instrument at 200 kV. X-ray photoelectron spectroscopy (XPS) spectra were obtained on a thermo ESCALAB 250XI X-ray photoelectron spectrometer. Crystallographic information was obtained using X-ray diffraction (XRD, Bruker-AXS D8 ADVANCE X-ray diffractometer). Fourier transform infrared (FT-IR) spectra were recorded on a Nicolet 6700 FT-IR spectrometer. Contact angles were measured with a KRÜSS DSA100 (KRÜSS, Germany) contact-angle test system at ambient temperature. The transmittance and reflectance spectra of PA66/PPy spheres were

obtained using a UV–visible spectrophotometer (Lambda 750 S UV Spectrometer). The infrared images were taken by a FLIR E8 infrared camera.

Evaluation of Solar Evaporation in Laboratory

The plastic container with a radius of 5 cm and a height of 6 cm was filled with simulated seawater (3.5 wt% NaCl solution). The PA66/$PPy_{0.001}$, PA66/$PPy_{0.01}$, PA66/$PPy_{0.05}$ PA66/$PPy_{0.1}$ and PA66/$PPy_{0.2}$ spherical evaporators were placed on the polystyrene (PS) foam with the tail contact with the bulk water, respectively. After stabilized for 20 min, a simulated sunlight (by 300 W xenon arc lamp) with a radiation intensity of 1 kW m^{-2} (1 sun) was used to drive steam generation at room temperature of 24 ^{o}C and humidity of 56%. The water mass change of a single spherical evaporator was monitored by an electrical balance and recorded every 10 min.

Solar Evaporation under Different Light Incident Angles

A single PA66/$PPy_{0.1}$ spherical evaporator was employed to evaluate the solar evaporation performance under the different light irradiation angles: 0°, 30°, 60° and 90° (the angle between light incident direction and horizontal plane) under the light intensity of 0.5, 1, 1.5 and 2 kW m^{-2}. The variation of sunlight incident angle was achieved by adjusting the angle of xenon lamps (Figure S16). PA66/PPy film wrapped a column PS foam with the wall connect with bulk simulated seawater at different light irradiation angles: 0°, 30°, 60° and 90° under the light intensity of 1 kW m^{-2}.

Photothermal Enhanced CO_2 Reduction

PA66/PPy/Cu_2O, PA66/Cu_2O photocatalytic spherical evaporators, pure Cu_2O powder, PA66 and PA66/PPy/Cu_2O films were conducted for CO and CH_4 evolution by placing the

photocatalysts on the surface of water in the quartz reactor. The reactor was purged with CO_2 for ten minutes to exhaust the air mixtures and CO_2 was filled in the device. Gas samples were analyzed using gas chromatography every hour. The purity of CO_2 used in this experiment is 99.999%.

Water Evaporation Outdoors

A model house of dimensions 40 × 30 × 35 cm (length × width × height) was used to evaluate outdoor evaporation rate. placed on a platform scale was made to load solar evaporators, which was placed in a natural environment to test the evaporation rate of the evaporator. The larger spherical evaporators with diameter of 7 cm under natural sunlight to generate steam was conducted on 19 Sept 2019 at Nanjing Tech University, Jiangpu campus. The spherical evaporators were pinned together and floated on the water which filled in the acrylic model house. The BSA224S electronic balance was used to measure the water loss. The GEAULIGHT CEL-NP2000 Solar Meter, FLIR E8 infrared camera and FLUKE F52-2 tester monitored the sunlight intensity, surface temperature of spherical evaporator and ambient air temperature, respectively. A 3 × 3 PA66/PPy spherical evaporator matrix was fabricated on a large PS foam board by the same method used in the laboratory. The evaporation performance was carried out from 10:30 am to 1 pm on 26 Jun 2020 at same venue.

2. Supplementary Figures

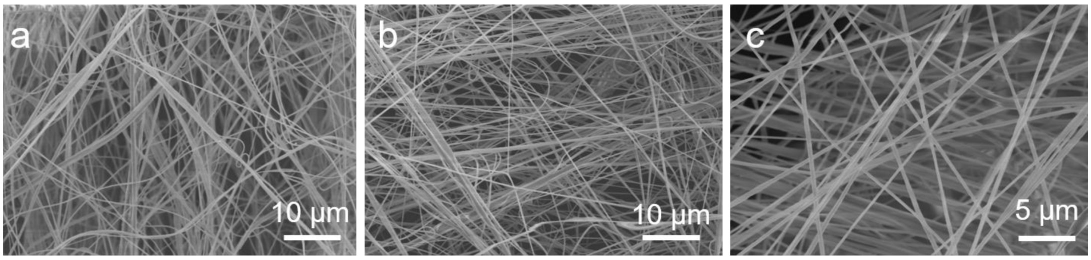

Figure S1. SEM images of PA66 NFs under the blow spinning conditions of solution concentration of 13%, flow rate of 5 mL h^{-1} and air pressure of a) 0.05, b) 0.1 and c) 0.2 MPa.

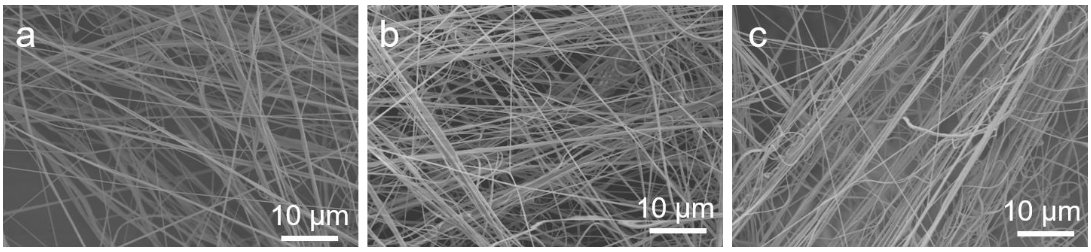

Figure S2. SEM images of PA66 NFs under the blow spinning conditions of solution concentration of 13%, flow rate of a) 4, b) 5 and c) 6 mL h^{-1} and air pressure of 0.1 MPa.

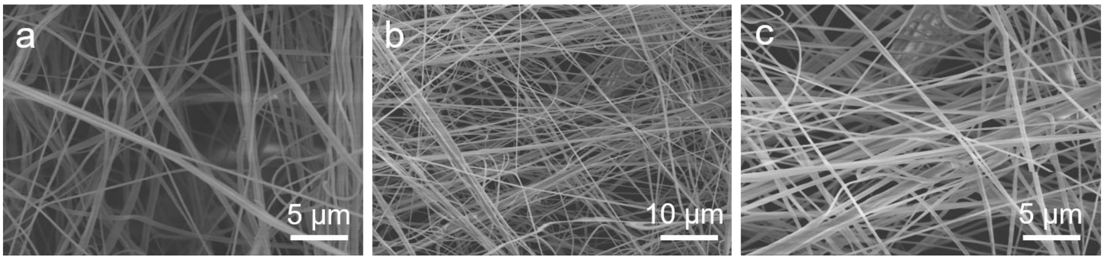

Figure S3. SEM images of PA66 NFs under the blow spinning conditions of solution concentration of a) 11%, b) 13% and c) 15%, flow rate of 5 mL h^{-1} and air pressure of 0.1 MPa.

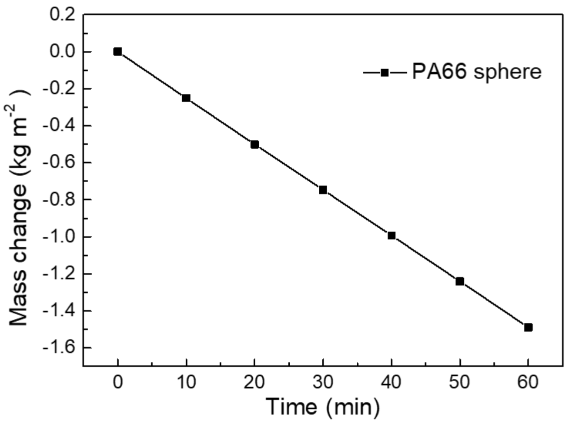

Figure S4. Evaporation mass change of the PA66 spherical evaporator.

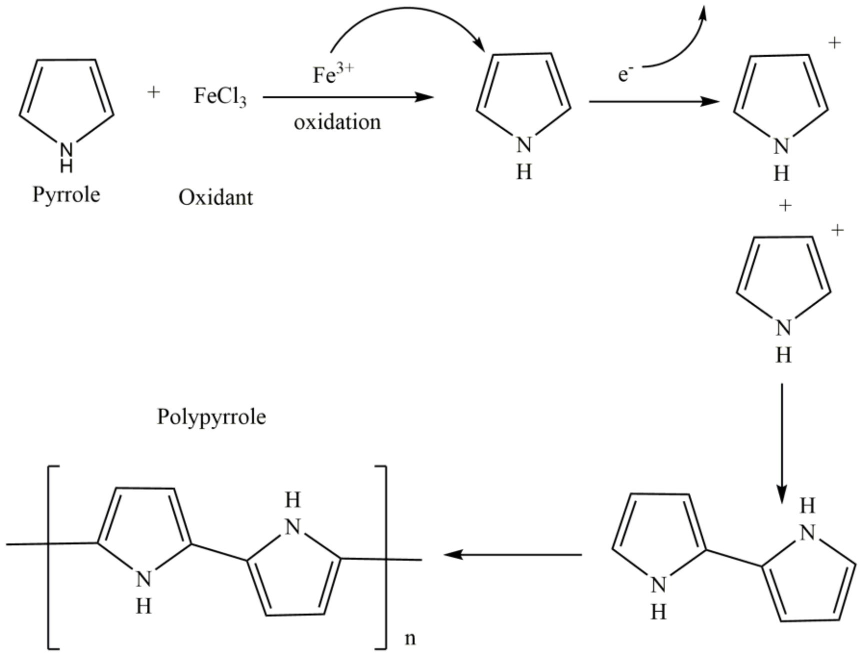

Figure S5. Synthetic route of PPy.[1, 2]

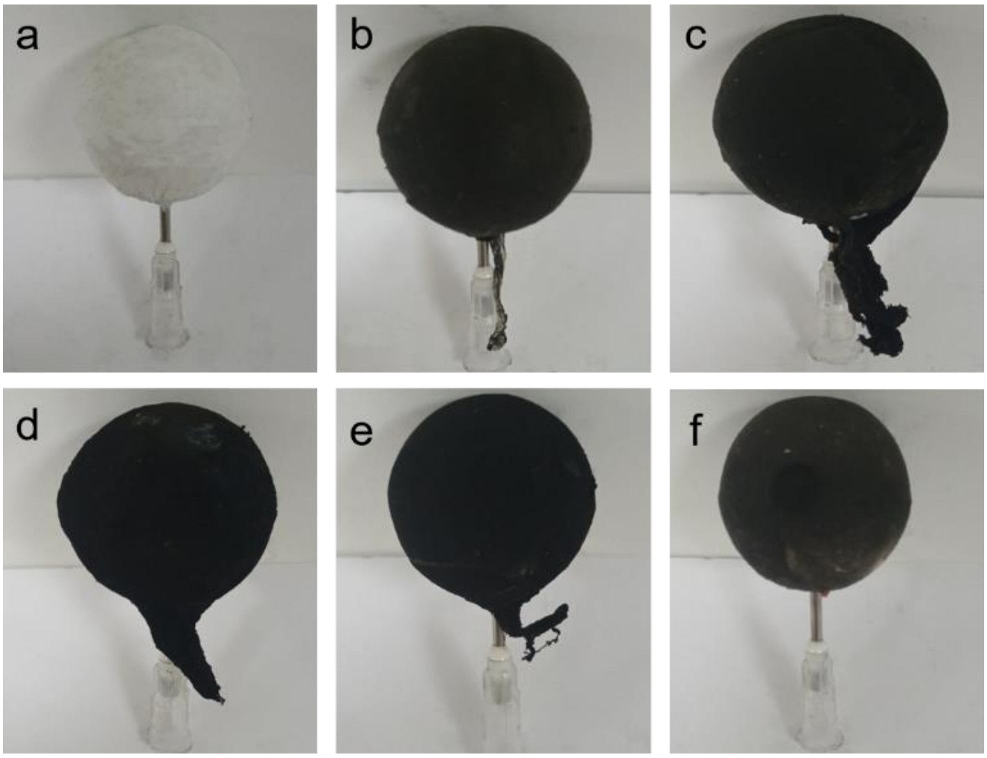

Figure S6. Photographs of a) PA66, b) PA66/$PPy_{0.001}$, c) PA66/$PPy_{0.01}$, d) PA66/$PPy_{0.05}$, e) PA66/$PPy_{0.1}$ and f) PA66/$PPy_{0.2}$ spheres.

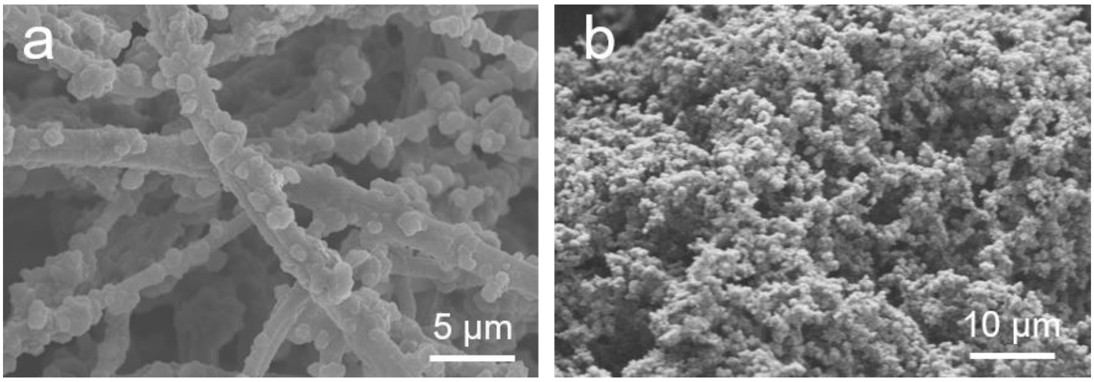

Figure S7. SEM images of a) PA66/$PPy_{0.001}$ and b) PA66/$PPy_{0.2}$ spheres.

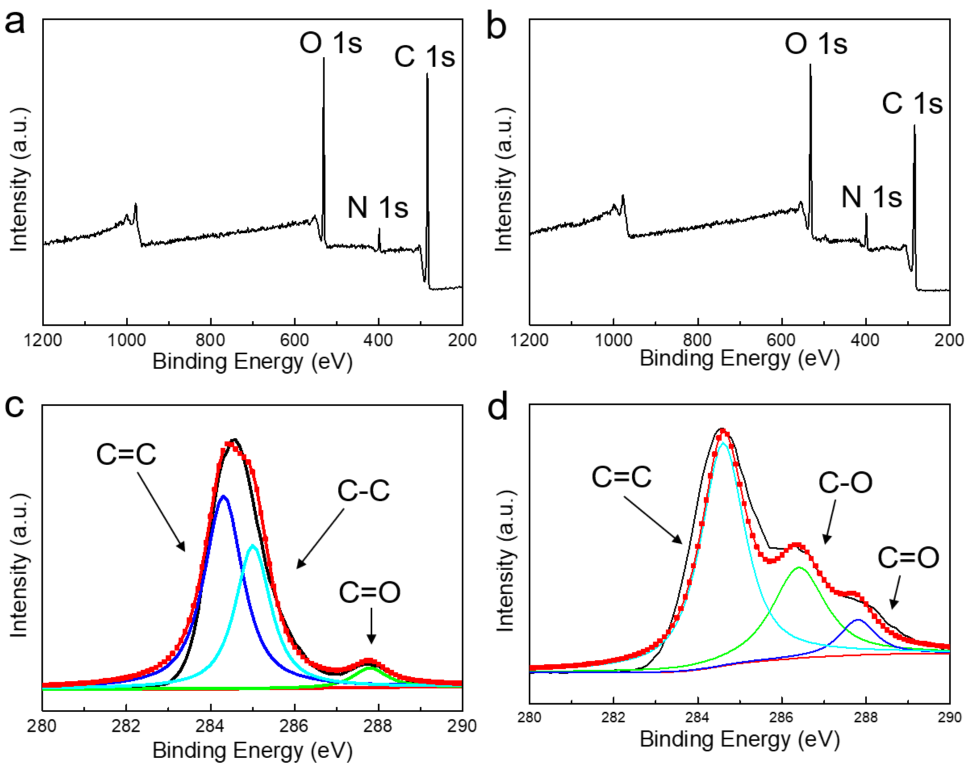

Figure S8. XPS spectra of a) PA66 and b) PA66/PPy NFs. XPS spectra for C1s of c) PA66 and d) PA66/PPy NFs.

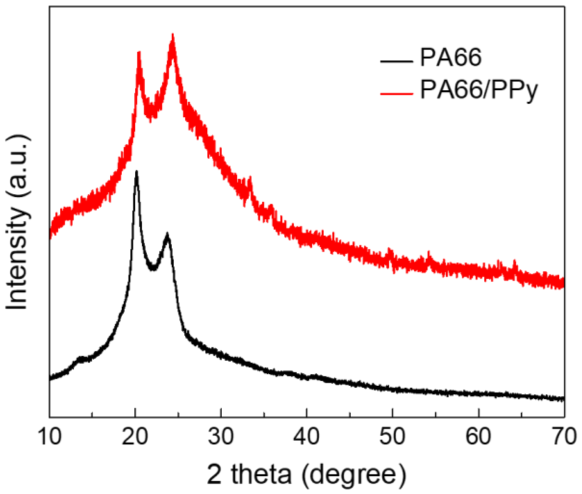

Figure S9. XRD patterns of PA66 and PA66/PPy NFs.

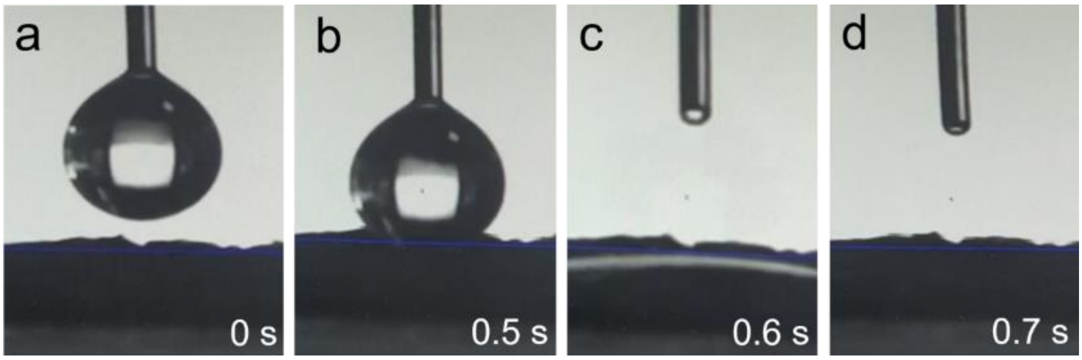

Figure S10. Photographs of water contact angle changes with time, a) 0, b) 0.5, c) 0.6 and d) 0.7 seconds.

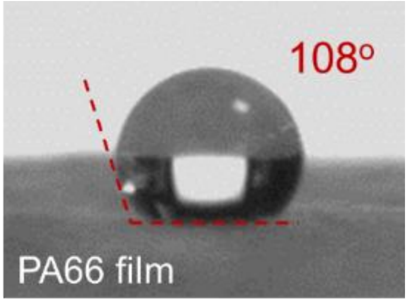

Figure S11. Photographs of water contact angle of PA66 film.

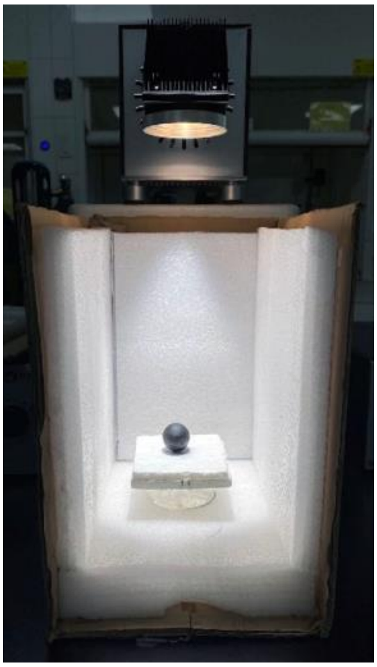

Figure S12. Photograph of water evaporation setup.

Calculation of photothermal conversion efficiency

The evaporation rate and light-to-vapor efficiency of water can be calculated by (1) and (2).[3-4]

$$m = \frac{\Delta m}{A} \quad (1)$$

$$\eta = \frac{m'(l_v + Q)}{P_{in}} \quad (2)$$

Where m (kg m^{-2} h^{-1}) is the water evaporation rate, Δm (kg) is the water mass change in 1 h. A (m^2) is the top projected plane area of the evaporator, η (%) is the efficiency of water evaporation. L_V is the latent heat of vaporization of water, normally taken to be 2.26 kJ g^{-1}. Q is the energy required for the evaporation system to heat from the initial temperature T_1 to the final temperature T_2. P_{in} is the light intensity on the evaporator.

According to the reported method, we measured the water vaporization enthalpy of PA66/PPy compoiste. The bulk water and PA66/PPy evaporator with the same surface area were placed in a closed space. The experiment was carried out in dark conditions under ambient pressure at room temperature. The mass change is recorded every hour and shown in Table S1.

Table S1. Cyclic test data of equivalent evaporation enthalpy experiment

Cycle	Evaporation rate of pure water (g m^{-2} h^{-1})	Evaporation of water in PA66/PPy (g m^{-2} h^{-1})	Room temperature (°C)	Evaporation rate ratio (water/(PA66/PPy))

1	126.2	101.5	20-21	0.8045
2	126.8	104.3	20-21	0.8248
3	130.1	110.9	22-24	0.8523
4	130.2	111.7	25-26	0.8542
5	134.6	113.5	25-26	0.8434
6	131.5	109.6	25-26	0.8335
7	131.2	105.2	22-23	0.8015
8	123.9	100.6	20-21	0.8116
9	122.5	99.4	19-20	0.823
10	126.9	101.6	19-20	0.8004

Using the reported vaporization enthalpy of pure water (~2256 J g^{-1}), the equivalent vaporization enthalpy of water in PA66/PPy can be calculated to be 1861 J g^{-1} according to the equation (3),

$$E_{PA66/PPy}=\frac{m_{water}}{m_{PA66/PPy}}E_{water} \quad (3)$$

Where $E_{PA66/PPy}$ is the equivalent vaporization enthalpy of water in PA66/PPy and $m_{PA66/PPy}$ is the solar evaporation rate of PA66/PPy.

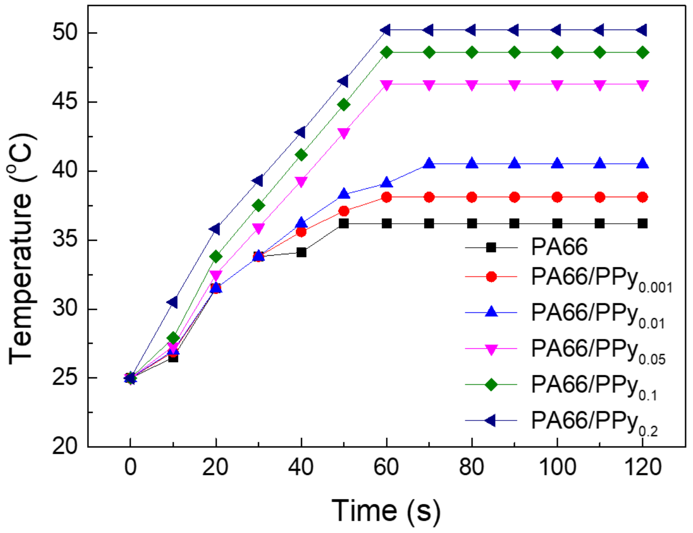

Figure S13. Surface temperature changes of PA66, PA66/PPy$_{0.001}$, PA66/PPy$_{0.01}$, PA66/PPy$_{0.05}$ PA66/PPy$_{0.1}$ and PA66/PPy$_{0.2}$ within the beginning 2 min.

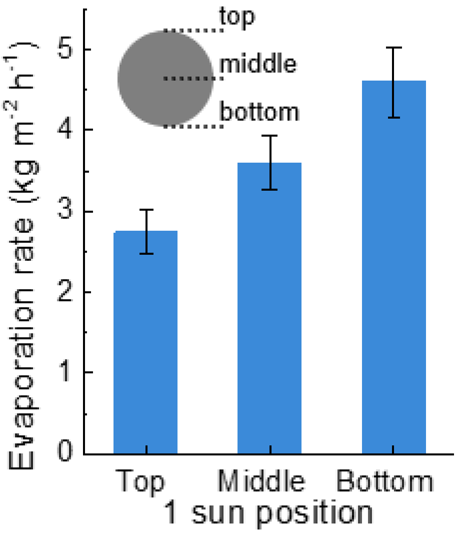

Figure S14. Evaporation rates as light density of 1 kW m^{-2} base on different positions.

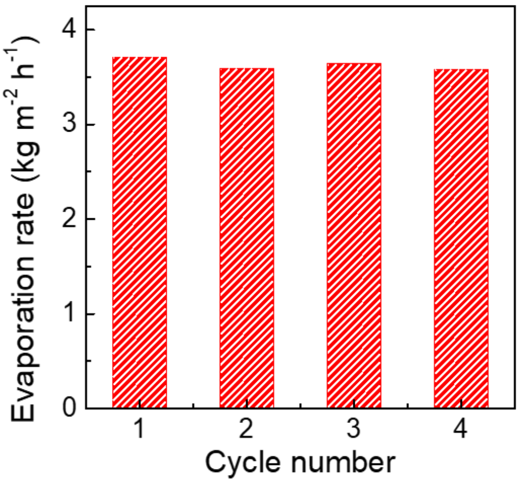

Figure S15. Stability and reusability of PA66/PPy$_{0.1}$ for steam generation.

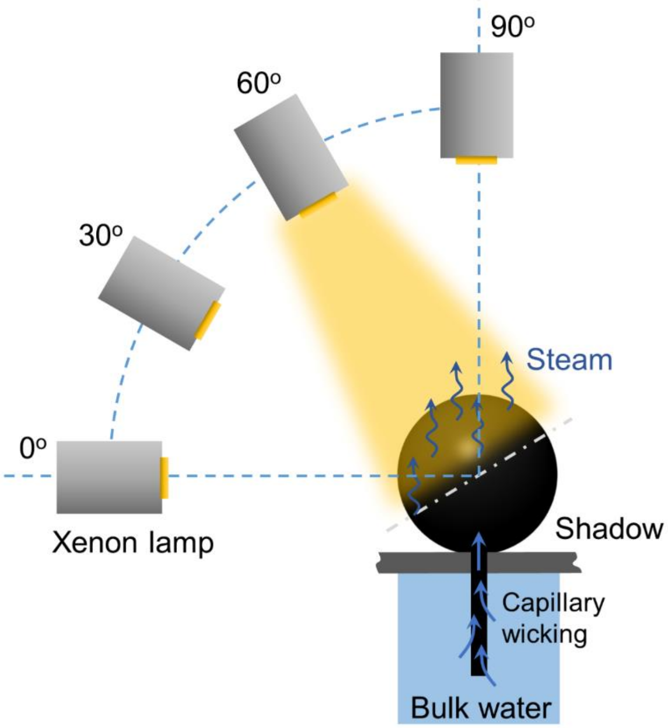

Figure S16. Schematic illustration of Xenon lamp position of the evaporation system under different light incident angles.

Table S2. Evaporation rates[1] of PA66/$PPy_{0.1}$ under different light intensities and angles

Light angle	Light intensity (kW m^{-2})			
	0.5	1	1.5	2
0°	2.04	3.33	4.30	5.02
30°	1.99	3.30	1.26	4.96
60°	2.04	3.17	4.23	5.03
90°	2.09	3.64	4.35	5.24

[1] Unit: kg m^{-2} h^{-1}

Table S3. Evaporation efficiencies of PA66/$PPy_{0.1}$ under different light intensities and angles

Light angle	Light intensity (kW m^{-2})			
	0.5	1	1.5	2
0°	244%	198%	171%	150%
30°	235%	198%	168%	148%
60°	244%	189%	170%	152%
90°	250%	217%	173%	156%

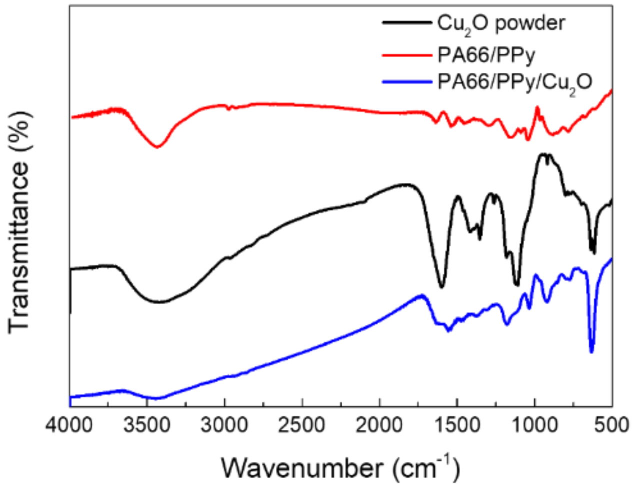

Figure S17. FTIR spectra of Cu_2O powder, PA66/PPy NFs and PA66/PPy/Cu_2O.

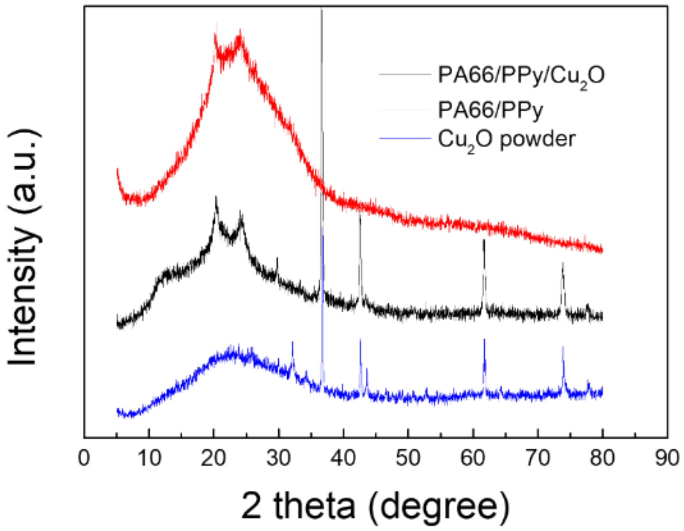

Figure S18. XRD spectra of Cu_2O powder, PA66/PPy NFs and PA66/PPy/Cu_2O.

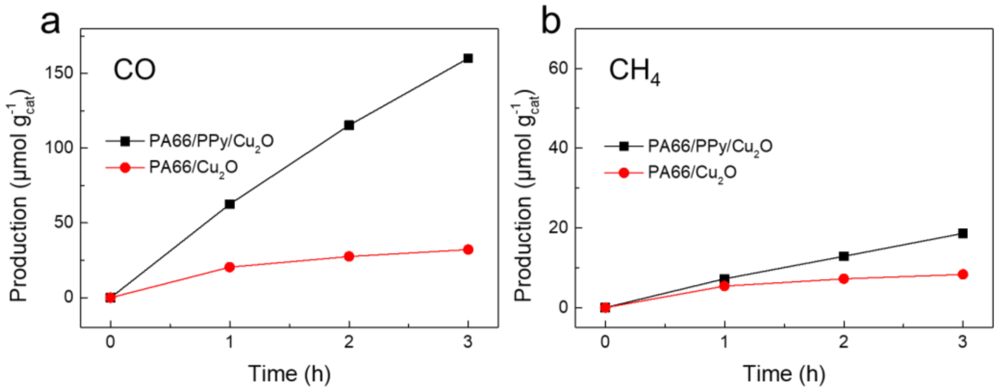

Figure S19. (a) CO and (b) CH_4 evolution by the spherical PA66/PPy/Cu_2O and PA66/Cu_2O evaporator under solar intensity of 1 kW m^{-2}.

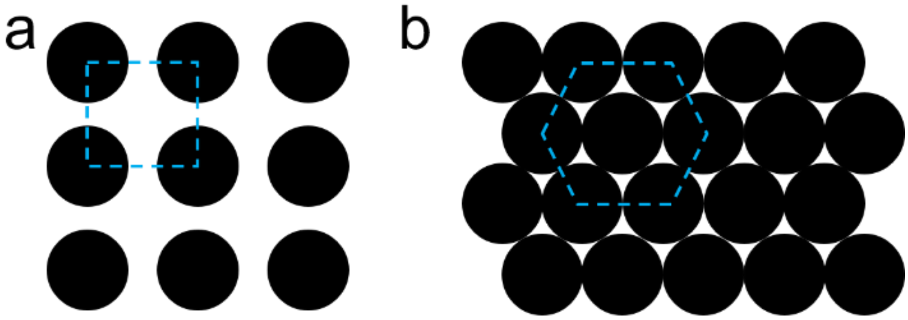

Figure S20. Schematic illustration of PA66/PPy spherical evaporator arranged in different configurations for outdoor solar evaporation test: a) quadrate and b) hexagonal configurations.

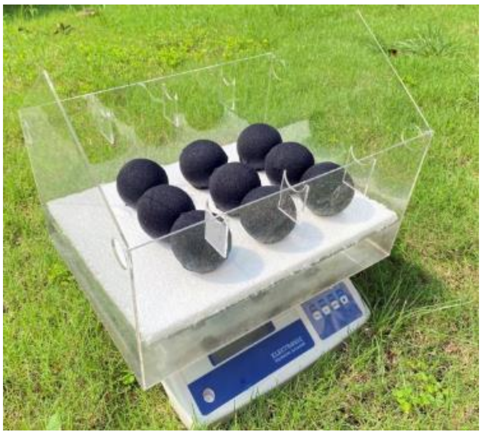

Figure S21. Photograph of assembled spherical evaporators.

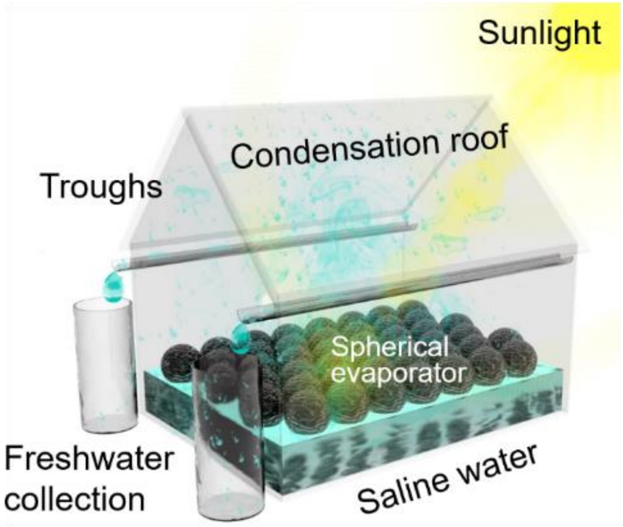

Figure S22. Schematic diagram of a model house solar evaporator assembled in hexagonal configuration.

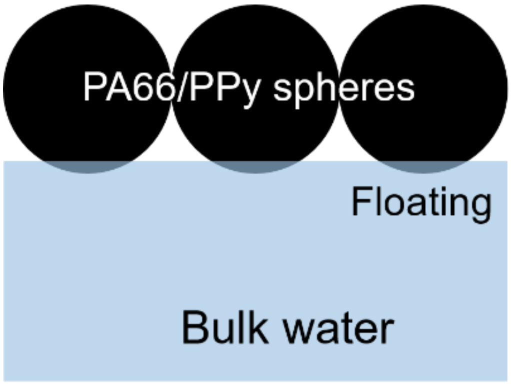

Figure S23. Schematic diagram of solar evaporators floating on water surface directly.

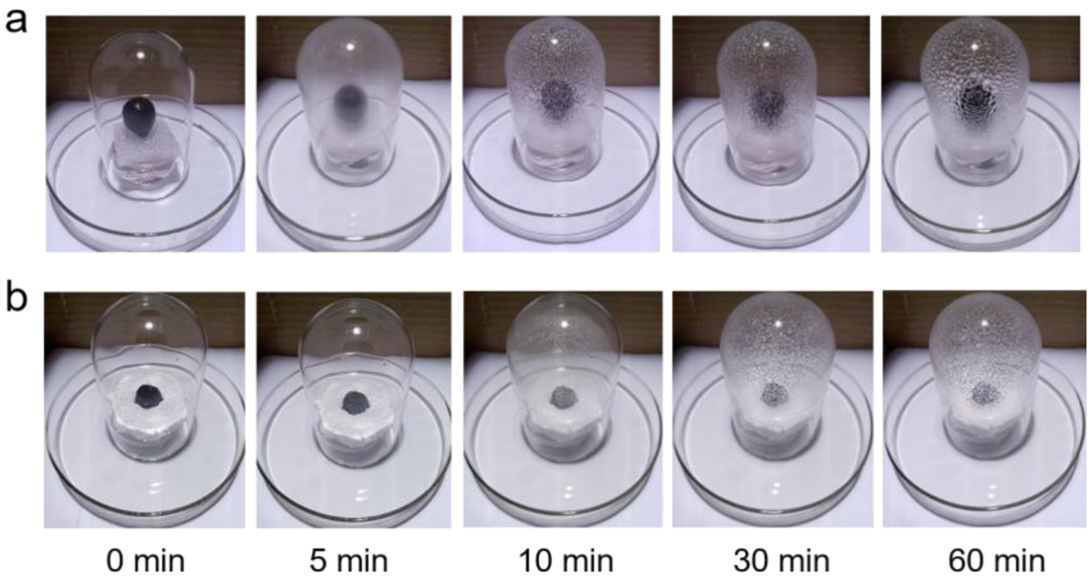

Figure S24. Photographs of the a) spherical and b) flat evaporators collect water within 60 min.

Table S4. Test data of water collection experiment

Cycle	Light to vapor rate (kg m^{-2} h^{-1})	Vapor to water rate (kg m^{-2} h^{-1})
1	3.61	2.21
2	3.57	2.13
3	3.66	2.31

Table S5. Outdoor water collection rate

Cycle	Water collection rate (kg m^{-2} day^{-1})	Average light intensity (kW m^{-2})
1	18.64	0.54
2	17.88	0.48
3	17.63	0.46

References

[1] J. Zhang, Y. Shi, Y. Ding, W. Zhang, G. Yu, *Nano Lett.* **2016**, *16*, 7276.

[2] H. Tang, J. Wang, H. Yin, H. Zhao, D. Wang, Z. Tang, *Adv. Mater.* **2015**, *27*, 1117.

[3] M. Gao, L. Zhu, C. K. N. Peh, G. W. Ho, *Energy Environ. Sci.* **2019**, *12*, 841.

[4] T. Li, Q. Fang, X. Xi, Y. Chen, F. Liu, *J. Mater. Chem. A* **2019**, *7*, 586.
